# Impact of novel coronavirus Disease-19 (COVID-19) pandemic in Italian pediatric emergency departments: a national survey

**DOI:** 10.1186/s13052-021-00996-8

**Published:** 2021-03-03

**Authors:** Valentina Talarico, Luciano Pinto, Gian Luigi Marseglia, Antonella Centonze, Concetta Cristofaro, Rocco Reina, Agostino Nocerino, Riccardo Lubrano, Stefania Zampogna, SIMEUP research group, Alberto Arrighini, Maria Antonietta Barbieri, Claudia Bondone, Silvia Bressan, Vincenza Corsi, Massimo Chiossi, Elisabetta Cortis, Laura Crespin, Antonio Cualbu, Liviana Da Dalt, Valeria De Donno, Maria De Filippo, Antonella Di Stefano, Pasquale Ferrante, Battista Guidi, Adima Lamborghini, Marcello Lanari, Cristina Malorgio, Sergio Manieri, Stefano Masi, Susanna Masiero, Beatrice Messini, Maria Pia Mirauda, Anna Maria Musolino, Rosaria Nigro, Giuseppe Parisi, Niccolò Parri, Massimo Pettoello-Mantovani, Flavio Quarantiello, Eduardo Ponticiello, Stefano Romero, Salvatore Savasta, Eurilla Sequi, Debora M. L. Simonetti, Eleonora Tappi, Antonio Francesco Urbino, Patrizia Vianelli, Tiziana Zangardi

**Affiliations:** 1 Department of Pediatric, “Pugliese-Ciaccio” Hospital, Viale Pio X, 88100 Catanzaro, Italy; 2 Italian Society of Pediatric Emergency Medicine, Naples, Italy; 3 Department of Pediatrics, Foundation IRCCS Policlinico “San Matteo” University of Pavia, Pavia, Italy; 4 Department of Pediatric Surgery, “Pugliese-Ciaccio” Hospital, Viale Pio X, Catanzaro, Italy; 5 Department of Law, History Economics and Social Science, Magna Graecia University, Catanzaro, Italy; 6 Department of Pediatrics, University Hospital of Udine, Udine, Italy; 7 Department of Pediatrics, “La Sapienza” University of Roma -Hospital of Latina, Roma, Italy

**Keywords:** Pediatric emergency, COVID-19, Personal protective equipment, Healthcare workers

## Abstract

**Background:**

Coronavirus Disease-19 (COVID-19) has rapidly become a pandemic emergency, distressing health systems in each affected country. Preparation strategies for managing this pandemic have been keys to face the COVID-19 surge all over the world and all levels of care.

**Materials and Methods:**

During the epidemic, the Italian society of pediatric emergency-urgency (SIMEUP) promoted a national survey aiming to evaluate preparedness and response of pediatric emergency departments (PED) critical in ensuring optimal management of COVID-19 cases.

**Results:**

Our results suggest that Italian PED have promptly set a proactive approach to the present emergency. 98.9% of the hospitals have defined special pathways and assistive protocols concerning the management of pediatric COVID-19 cases. The highest percentage of application of the measures for preventive and protective for COVID-19 concerned the use of personal protective equipments.

**Conclusions:**

Results show that the following measures for pediatric patients, admitted in PED, have been promptly implemented throughout the whole country: eg. use of protective devices, pre-triage of patients accessing the hospital. Despite COVID-19 being a new threat, we have shown that by developing an easy-to-follow decision algorithm and clear plans for the interventional platform teams, we can ensure optimal health care workers and patients’ safety.

## Background

A novel coronavirus belonging to beta-coronavirus genera (named as 2019-nCoV by World Health Organization [WHO]) was identified as the causative agent associated with a cluster of cases of pneumonia detected in Wuhan City by Chinese authorities on 7 January [1, 2]. On January 2, 2020, the first 2 cases, two Chinese tourists, were identified in Italy; on January 30, 2020, WHO re-evaluated the potential effects of 2019-nCoV infection in global public health, and declared this epidemic as a “Public Health Emergency of International Concern (PHEIC)”. Since the discovery of 2019-nCoV, the virus has been rapidly spreading all over the word [3, 4]. Infected children might appear asymptomatic or have mild clinical manifestations: fever, dry cough, and fatigue, and few have upper respiratory symptoms including nasal congestion and running nose; some patients presented with gastrointestinal symptoms including abdominal discomfort, nausea, vomiting, abdominal pain, and diarrhea [5–8]. However, cases of major systemic inflammation (Kawasaki-Like Hyperinflammatory Syndrome) have been reported in children infected with coronavirus [9]. A study that involved a cohort of 100 Italian children younger than 18 years of age (*The Coronavirus Infection in Pediatric Emergency Departments*, CONFIDENCE) who were assessed between March 3 and March 27 in 17 Pediatric Emergency Departments (PED) (median age 3,3 years) observed that common symptoms were cough (in 44% of the patients) and no feeding or difficulty feeding (in 23%). A total of 54 children (54%) had a temperature of at least 37.6 °C and 12 (12%) appeared ill, and 38 children (38%) were admitted to the hospital; 20 of them had moderate or severe disease (19 and 1 respectively) [10]. Another study that involved 102 centers from 18 countries found a wide variation on personal protective equipment (PPE) use at pre**-**triage and for patient assessment [11]. Preparation strategies for managing this pandemic have been needed all over the world and in all the hospitals. Reallocation of resources in the management of this crisis implied careful planning, the interruption of scheduled hospitalizations, and the training of health workers. Strict adherence to infection control such as PEE and disinfection are the keys to contain the transmission of the disease. In particular, the key interventions of the PED for the management of this pandemic have been different. Mandatory hospitalization in isolation wards for all suspected cases presenting to the PED and the segregation of PED into high-risk, intermediate-risk, and low-risk areas are crucial elements for reduction the possible rate of infection [12].

The provision of qualified PPE to health care workers plays an essential role in *preventing* occupational exposure and infection. US Centers for Disease Control and Prevention for COVID-19 infection control of healthcare personnel recommended gloves, gowns, respiratory protection, and eye protection as standardized PPE [13].

The Italian society of pediatric emergency-urgency (SIMEUP) brings together various professional figures who manage children in the acute care setting, belonging to both general and pediatric hospitals. During the COVID-19 epidemic in Italy, the SIMEUP promoted a national survey aiming to evaluate the impact of COVID-19 on the clinical acute pediatric practice and the strategies activated at the hospital level for the management, infectious containment and reorganization of the work in the acute care pediatric setting during this pandemic.

## Material and methods

We carried out an observational study on organizational changes that took place in Italy pediatric departments following the COVID-19 pandemic. The survey was launched online on 1st April 2020 and closed on 30th April 2020. It included a total of 15 questions, divided into some large groups. The key points of the survey were:

- implementation of organizational protocol for the management of “suspected” or “confirmed” cases of pediatric COVID-19. In particular was asked whether there were specific regional, or local protocols for the management of pediatric COVID cases. A particular question was about there were hospital care pathways on specific aspects of the management of a COVID case (e.g. swab execution modality, bronchial or aspirate washing, hospitalization or transfer modalities, specific paths for newborns and/or pregnant women)
- re-organization of spaces: we asked about the creation of dedicated pre-triage, with trained and qualified personnel, in particular if there were pediatric triages; the creation of waiting zones dedicated to children.
- use of PPE, before and after the pandemic, and ease of their procurement; we used a 5-point Linkert scale (1: never to 5: always) about the use of different PPE (in particular gloves, gowns, glasses, various type of facial masks, head cap, protective visor and shoe covers) and about the capacity of procurement PPE before and during the pandemic.
- modification of the emergency and planned work activities. The survey asked the number of visits to PE and the number of hospitalized children, during the month of March 2020 compared to the same period in the past year.

The questionnaire was sent to all SIMEUP members, made up of medical and nursing staff, coordinated by the SIMEUP regional presidents.

Descriptive statistics were used to analyze the data through the MedCalc Software (Ltd di Ostenda, Belgio).

## Results

**Fig. 1:**
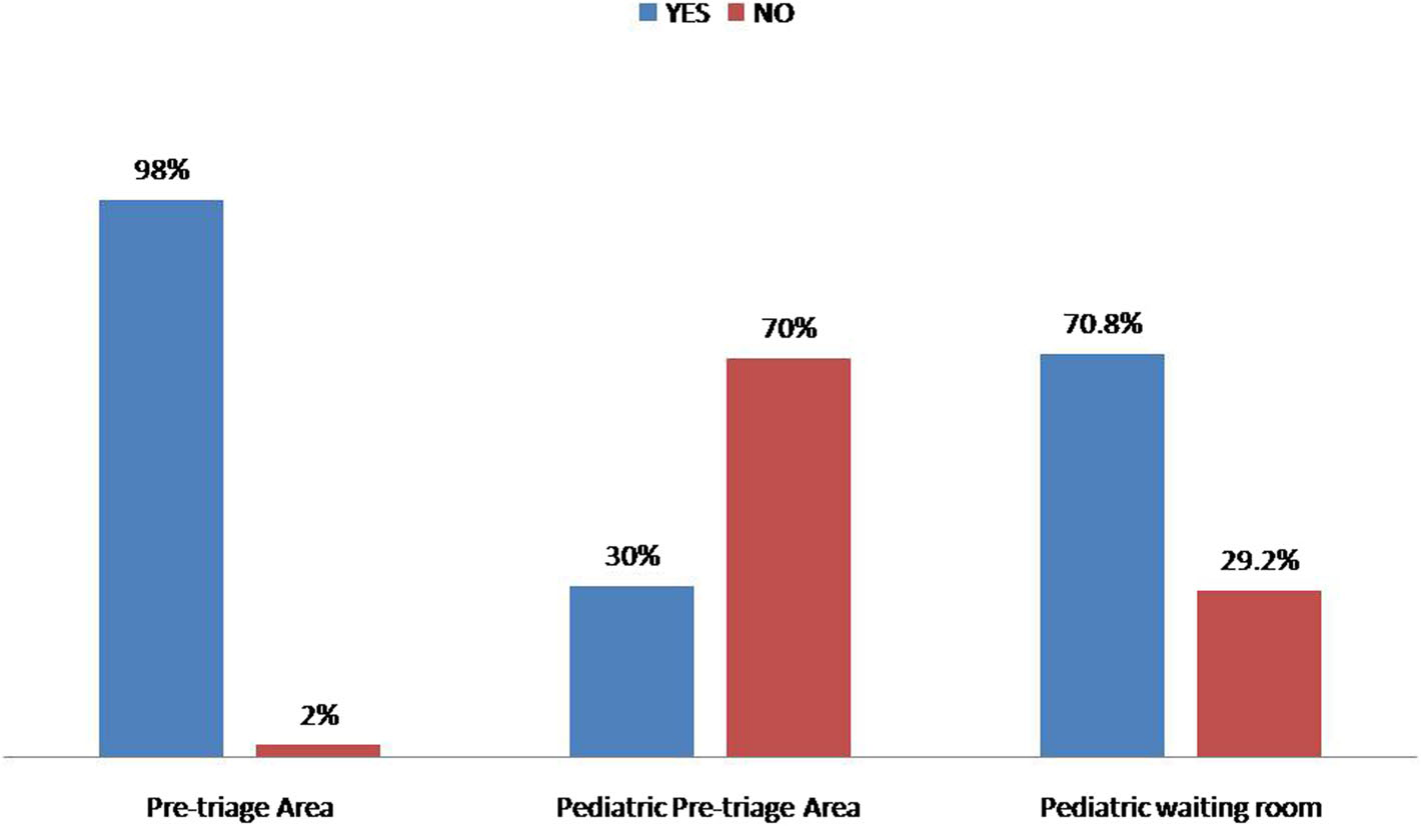
Creation of specific areas in PED

During the study period, we received the questionnaire replies from about 94 hospitals, of 20 Italian Region. The answers came from different health professionals (110 doctors, 30 nurses). The creation of specific organizational plans defined at a regional level, for the management of pediatric COVID-19 cases, occurred in about 63.8% Italian regions. However, almost 96.6% of the individual hospitals contributed to the creation of special pathways and assistive protocols concerning the management of COVID-19 cases for both adult and pediatric age. The most important structural modification consisted in the creation of pre-triage areas, as described in Fig. 1. About 98% of the hospitals has created a special pre-triage, but only in 30% of cases, it was dedicated to the pediatric age.

**Fig. 2:**
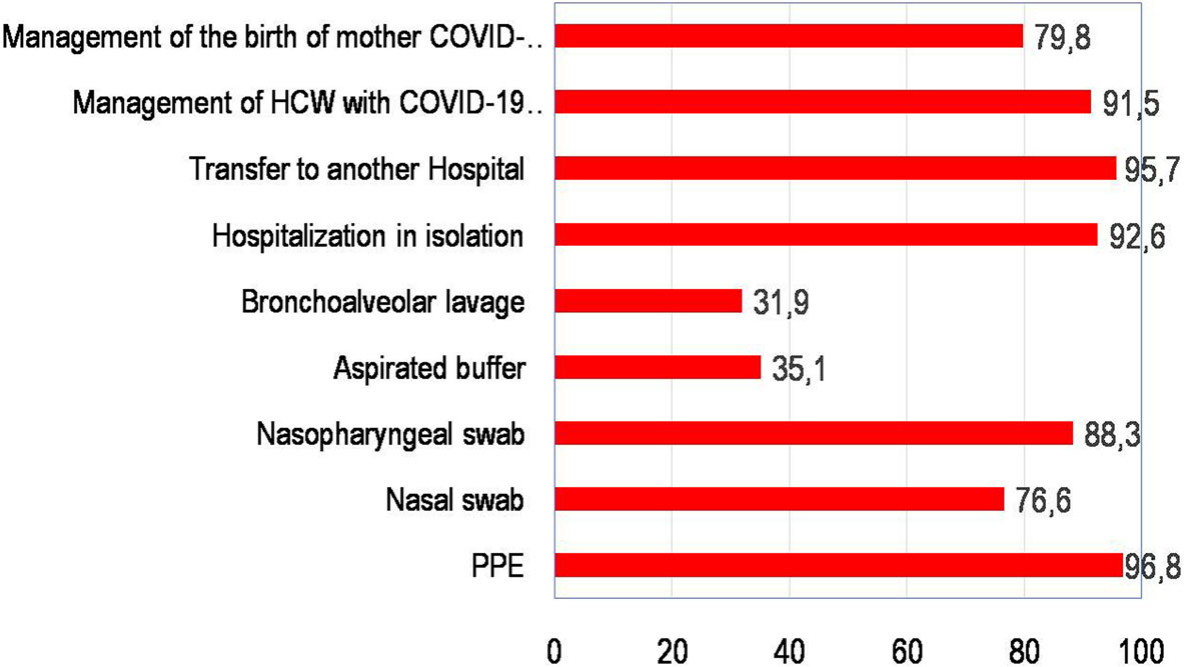
Application of protective and preventive measures for COVID-19

The hospital directives mainly concerned the use of PPE (95%), the methods of performing the nasopharyngeal swab (85.71%), the hospitalization of infectious subjects (84.29) and their transfer to other hospitals (Fig. 2). Interestingly, over 75% of hospitals have defined their own care pathway for the management of a mother or newborn infected with COVID 19.

About PPE, Table 1 shows the changes in the usual use of PPE before and after the pandemic. A positive trend is evident in the use of all devices, in particular the surgical mask (222.54%). This trend of greatest use is uniform for both medical and nursing figures (Table 2). The percentage of use of PPE in the “Suspected” and “Confirmed” cases of COVID-19 was correctly distributed throughout the national territory (Table 3).

**Table 1:** Usual use of PPE before and after the onset of COVID-19 min 1(never)- max 5 (always)

	Double gloves	Disposable gown	Waterproof gown	Surgical face mask	FFP2 without valve	FFP2 with valve	FFP3	Goggles	Surgical head cap	Protective Visor	Shoe covers
Before COVID-19	2,78	1,93	1,19	1,42	2,26	1,27	1,31	1,74	1,58	1,72	1,37
After COVID-19	4,37	3,83	2,84	4,58	2,31	2,71	2,13	3,40	3,54	3,13	2,82
Variation (%)	1,59	1,91	1,65	3,16	0,05	1,44	0,81	1,66	1,96	1,41	1,45

*PPE* personal protective equipment

**Table 2:** Usual use of PPE before and after the onset of COVID-19 for different healthcare workers min 1 (never)- max 5 (always)

PPE	Nurse				Doctor			
	Before		After		Before		After	
	Media	SD	Media	SD	Media	SD	Media	SD
Double gloves	**3,88**	1,09	**4,40**	1,15	**2,50**	1,07	**4,39**	0,96
Disposable gown	**2,16**	1,03	**3,80**	1,32	**1,87**	1,04	**3,86**	1,20
Surgical mask	**1,68**	1,04	**4,28**	1,40	**1,34**	0,74	**4,72**	0,83
Waterproof gown	**1,25**	0,64	**3,17**	1,47	**1,16**	0,41	**2,78**	1,33
FFP2 without valve	**2,39**	1,08	**2,72**	1,28	**2,23**	1,03	**2,23**	1,21
FFP2 with valve	**1,30**	0,63	**3,04**	1,55	**1,25**	0,56	**2,64**	1,35
FFP3	**1,52**	0,85	**2,64**	1,41	**1,23**	0,55	**2,02**	1,26
Surgical head cap	**2,12**	1,01	**3,92**	1,35	**1,64**	0,99	**3,43**	1,31
Glasses	**1,88**	0,93	**3,52**	1,29	**1,48**	0,88	**3,39**	1,28
Protective visor	**1,59**	0,85	**3,23**	1,38	**1,74**	1,10	**3,10**	1,43
Shoe covers	**1,41**	0,85	**3,27**	1,42	**1,34**	0,80	**2,73**	1,40

*PPE* personal protective equipment, *SD* standard deviation

**Table 3:** Use of PPE in “suspected” and confirmed” case of COVID-19

CASE	Double gloves	Disposable gown	Waterproof gown	Surgical facial mask	FFP2 without valve	FFP2 with valve	FFP3	Glasses	Surgical head cap	Protective Visor	Shoe covers
Suspected	96,24	83,33	85,04	71,82	56,64	73,83	41,24	92,13	94,49	82,79	76,27
Confirmed	99,20	67,96	96,72	51,52	58,76	62,22	58,42	89,74	98,33	90,08	89,26
*Variation (%)*	*2,96*	*−15,37*	*11,68*	*−20,30*	*2,13*	*−11,61*	*17,18*	*−2,38*	*3,85*	*7,30*	*12,99*

*PPE* personal protective equipment

The ability to find PPE in the daily work of health professionals before and after the pandemic is represented in Table 4.

**Table 4:** Difficulties in procuring protective equipment before and after the onset of COVID-19

	Gloves	Facial mask	Disposable gown	Glasses	Disinfectants
After COVID-19	3,98	3,22	3,44	3,53	3,75
Before COVID-19	4,18	3,93	3,97	3,76	4,05
Variation	−0,21	−0,71	−0,53	−0,23	−0,30
*Percentage of reduction (%)*	*4,96*	*18,06*	*13,38*	*6,18*	*7,36*

The comparison of PED visits and the number of admissions between March 2019 and 2020, showed that there was an overall reduction of 75.58% for emergency visits (*− 72.566*) and 68.42% for admissions (*− 8.188*). In 15 out of 94 hospitals, pediatric beds were used to care for adult infected by Covid-19 patients during that time.

## Discussion

According to the Statistical Yearbook of the National Health System (published in 2019), in Italy there were 518 public hospital structures, 414 with a General Emergency Room (79.9%) and 90 with a Pediatric Emergency Room (17.5%) The survey was attended by 94 hospitals representing all the Italian regions (with the exception of the autonomous province of Trento) [14]. The results of the study suggest how the Italian PEDs have promptly taken action to manage the pandemic with standardized pathways and protocols.

World literature data shows that one of the most important actions that have been activated to manage the emergency from coronavirus has been to take urgent actions to reorganize health services and protect health workers to take care of patients with COVID-19 in safety and save lives. The COVID-19 outbreak has highlighted a gap between infectious disease healthcare and epidemiologist advice for preventing the spread of the disease versus the actions taken by the state authorities that in many cases have been too late and inadequate [15].

The hospital preparedness for epidemics and pandemics of COVID-19 has taught us several lessons, which deviate from the classical safety and protection protocols for healthcare personnel those who remain on the frontline, fighting against the further spread and tirelessly treating those who are diseased [15]. Due to a lack of sufficient awareness of the COVID-19 in the early stages of the epidemic, some healthcare workers (HCWs) have been infected [16].

Following the Chinese model, containment measures to reduce the risk of COVID-19 in Italy have been promptly activated and implemented [17]. Due to the necessity to set up emergency management protocols for the prevention and control novel coronavirus (2019-nCoV) infection spread, various hospitals have completely reorganized their work and space. As highlighted in our work, almost all Italian hospitals have defined specific pathways for pediatric cases of COVID-19, with the creation of dedicated pre-triage structures, sometimes only for children (30%).

Numerous pediatric departments, both in Italy and in the rest of the world, had to initiate a series of structural and organizational changes to manage this pandemic. For example, the American pediatric response to the COVID-19 pandemic implemented change in 4 domains: physical space, clinical services, staffing models, and leadership. The goal was to continue to provide outstanding and safe pediatric care while at the same time supporting the adult emergency COVID-19 crisis [18].

To prevent the spread of COVID-19 to healthcare workers, additional infection control measures were implemented. Mask fitting exercises and PPE training was provided to all healthcare personnel. The most important PPE includes an N95 mask, eye protection with goggles or an eye shield, gown, and gloves [19].

The type and amount of PPE that should be used when treating a patient with COVID19 varies based on clinical job and setting [20]. For HCWs providing direct inpatient care for patients with COVID-19, a medical mask, gown, gloves, and eye protection in the form of goggles or a face shield should be used. If aerosol-generating procedures are being performed, healthcare workers should also wear an apron and use an N95 respirator in the place of a surgical mask [21]. All these indications have been respected in all pediatric departments in our nation; as can be seen in Table 1, there has been an increase in the use of PPE after the pandemic in all HCWs.

This data, especially in the early stages of the pandemic, inevitably led, as emerged in our work, to a significant difficulty in the procurement of PPE.

All these structural changes in hospitals, and the increase in the use of PPE, inevitably had a major economic impact. Tassa Tan-Torres et al. have recently published a study on the expected economic needs for an adequate response to covid-19. They conclude that the costs of a response to COVID-19 in the healthcare sector will increase considerably, particularly if transmission increases, but putting in place early and comprehensive measures to limit the further spread of the virus will conserve resourced and support the response [22].

Finally, another important fact that emerges from our investigation was the reduction of pediatric emergency visits hospitalizations across the nation. Our results show a reduction of approximately 70% for both visits to pediatric emergency departments and in the number of hospitalizations. However, as highlighted in another study, given the reduction rate in the number of accesses, the children were referred to the ED more frequently by physicians or the out-of-hospital emergency system, and for reasons that were more frequently urgent [23]. The substantial decreases in pediatric care access in Italy might reflect the scarcity of available resources due to pandemic-related redistribution, or reticence on the part of parents and caregivers to risk exposure to severe acute respiratory syndrome coronavirus-2 (SARS-CoV-2) in a health-care setting, in addition to lower rates of acute infections and trauma [24]. There is a need to prevent delays in accessing hospital care and to increase the provision of high-quality coordinated care by health-care providers.

Both of these aspects should be considered as part of the overall public health impact of the COVID-19 pandemic, as evident in other epidemics [25] and must be adequately monitored. Both the general population and health-care workers need clear guidance and information. Specifically, parents should be made fully aware that the risks of delayed access to hospital care for emergency conditions can be much higher than those posed by COVID-19.

The authors acknowledge the non-uniformity and selection bias limitations associated with non-random convenience sampling in surveys, while recognizing the generalizability and internal and external validity of the survey approach.

The sample size is relatively low and there is likely to be variability in the practices observed between different health professionals, hospitals, and regions with varying prevalence. However, we believe that the results obtained may be explanatory of the real situation in which the PED lived during the first months of the COVID 19 pandemic wave.

## Conclusions

Although the pediatric age was not severely affected by SARS-CoV2, it was not completely spared. Initial data from the Chinese Center for Disease Control and Prevention which evaluated case series of 2143 pediatric patients with COVID-19 showed that although the majority of cases had mild or no symptoms, severe and critical cases were present in 10.6, and 7.3%, for the age groups < 1 and 1–4 years [26], and a large US study revealed that children may be a potential source of contagion in the SARS-CoV-2 pandemic even in the absence of symptoms [27]. Two Italian descriptive studies during the first months of the pandemic reported respectively that 8.5% had moderate disease, 8.5% had a severe disease and 6.9% had a critical presentation [28] and that 43% had been hospitalized, confirming that, although severe cases are rare, children may represent a source of viral transmission [29].

This new global challenge represented by COVID-19 has proven how fundamental it is to strengthen health systems, but also the ability to produce rapid reorganization of the provision of services to respond to COVID-19, while at the same time the basic essential services worked through the continuous care so that nobody was left behind. The prompt activation and implementation of containment measures to reduce the risk of COVID-19 in Italy, together with the attention and reorganization of Hospitals and Departments, further reduced the risk of transmission of the infection.

As we refined our understanding of COVID-19, a high degree of active surveillance in the community and dynamic reassessment of pediatric department workflow processes, in conjunction with the hospital and nationwide public health response, helped us better manage this ongoing pandemic and prepare for the next.

## Data Availability

This manuscript has not been published and is not under consideration for publication elsewhere. The data presented are original.

## Abbreviations

COVID-19: Coronavirus Disease-19
HCWs: healthcare workers
PEE: personal protective equipment
PED: pediatric emergency departments
SARS-CoV-2: severe acute respiratory syndrome coronavirus 2
SIMEUP: Italian society of pediatric emergency-urgency
